# A CMOS Pressure Sensor Tag Chip for Passive Wireless Applications

**DOI:** 10.3390/s150306872

**Published:** 2015-03-23

**Authors:** Fangming Deng, Yigang He, Bing Li, Lei Zuo, Xiang Wu, Zhihui Fu

**Affiliations:** 1 School of Electrical and Electronic Engineering, East China Jiao Tong University, Nanchang 330013, China; E-Mails: zgxiangyu@163.com (X.W.); fuzhihui@sohu.com (Z.F.); 2 School of Electrical Engineering and Automation, Hefei University of Technology, Hefei 230009, China; E-Mails: 18655136887@163.com (Y.H.); libinghnu@163.com (B.L.); 18655118171@126.com (L.Z.)

**Keywords:** pressure sensor, RFID technology, CMOS process, capacitive sensor interface, rectifier

## Abstract

This paper presents a novel monolithic pressure sensor tag for passive wireless applications. The proposed pressure sensor tag is based on an ultra-high frequency RFID system. The pressure sensor element is implemented in the 0.18 µm CMOS process and the membrane gap is formed by sacrificial layer release, resulting in a sensitivity of 1.2 fF/kPa within the range from 0 to 600 kPa. A three-stage rectifier adopts a chain of auxiliary floating rectifier cells to boost the gate voltage of the switching transistors, resulting in a power conversion efficiency of 53% at the low input power of −20 dBm. The capacitive sensor interface, using phase-locked loop archietcture, employs fully-digital blocks, which results in a 7.4 bits resolution and 0.8 µW power dissipation at 0.8 V supply voltage. The proposed passive wireless pressure sensor tag costs a total 3.2 µW power dissipation.

## 1. Introduction

Micro-Electro-Mechanical Systems (MEMS) pressure sensors have been widely applied in consumer electronics, automotive systems, environmental monitoring, medical diagnostics, *etc.* [1]. According to the sensing principle, MEMS pressure sensors can be divided into three types, including piezoresistive pressure sensors, capacitive pressure sensors and resonant pressure sensors. Compared with other pressure sensors, the capacitive pressure sensor shows some significant advantages such as high sensitivity, low power, low noise and low temperature drift coefficients [2]. Recently different types of capacitive pressure sensor were reported [1,2,3,4,5], but these designs don’t integrate any signal processing circuits, resulting in increasing parasitic capacitance and lowering sensor performance. 

Radio Frequency IDentification (RFID), as a wireless automatic identification technology, is widely applied in traffic management, logistics transportation, medicine management, food production, *etc.* [6]. The passive RFID tag offers several advantages such as battery-less operation, wireless communication, high flexibility, low cost and fast deployment, which all result in its extensive applications in commercial use [7]. The passive RFID tag collects the radiation energy from the RFID reader as its power supply. Hence, the power dissipation of passive RFID tag, which determines the maximum reading distance of the RFID reader, is crucial for the design of passive RFID tags. Recently, with the rapid development of the Internet of Things and sensor technology, research on adding sensing functionality to RFID tag has become a hot topic [8,9,10]. This smart RFID sensing tag not only extends the RFID application fiels, but also contributes to reduce the fabrication cost of RFID systems. Therefore, the design of these RFID tags for sensor applications has to meet low power requirements. 

The paper presents a novel Complementary Metal Oxide Semiconductor (CMOS) pressure sensor tag chip for passive wireless applications. The rest of the paper is organized as follows: Section 2 introduces the system design of the proposed passive wireless pressure sensor tag chip. The key block designs of this work, including pressure sensor, rectifier and sensor interface, are illustrated in details in Section 3, Section 4 and Section 5. Section 6 presents the measurement results and some conclusions are given in Section 7.

## 2. System Design

The operating frequency plays a very important role in RFID systems. In general, the operating frequency defines the data rate between the tag and the reader. Lower operating frequency usually means a slower data rate. In addition to data rate, operating frequency also determines the tag size. The RFID operating frequency mainly falls into three categories, namely Low Frequency (LF—125 kHz), High Frequency (HF—13.56 MHz), Ultra-High Frequency (UHF—900 MHz) [11]. UHF—900 MHz RFID systems are a better solution for next generation auto-ID applications because they have fast transmission throughput and long distance communication.

**Figure 1:**
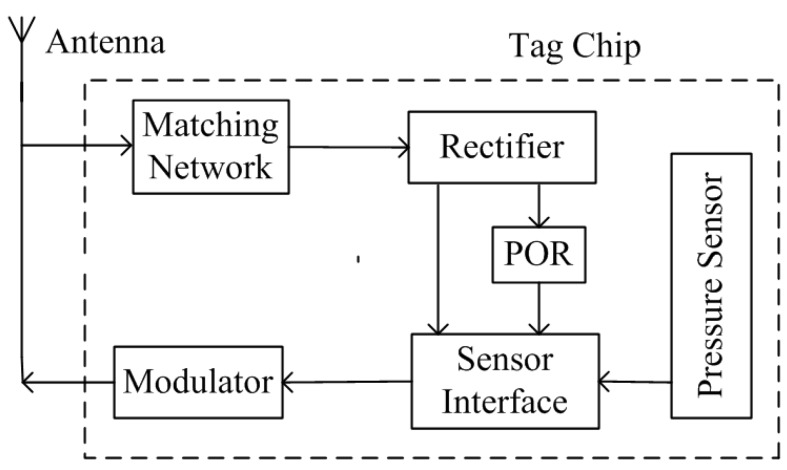
Block diagram of the proposed passive wireless pressure sensor tag.

Figure 1 shows the block diagram of the proposed passive wireless pressure sensor tag [12]. Those blocks, except the antenna, are integrated in a single-chip. The tag antenna, which is matched with the tag chip through a matching network, receives the electromagnetic wave from the RFID reader. The rectifier converts the received Radio Frequency (RF) signal to a stable supply voltage for the remainder of the circuitry. A voltage regulator is not included in the tag because the capacitive sensor interface is immune to the changes in supply voltage value as discussed in detail in Section 5. When the output of the rectifier settles, the Power-On-Reset (POR) block generates a reset signal for the sensor interface. There is no receiver block in the tag, which means that the tag will operate without any addressing as long as enough voltage is generated from the incoming electromagnetic wave.

**Figure 2:**
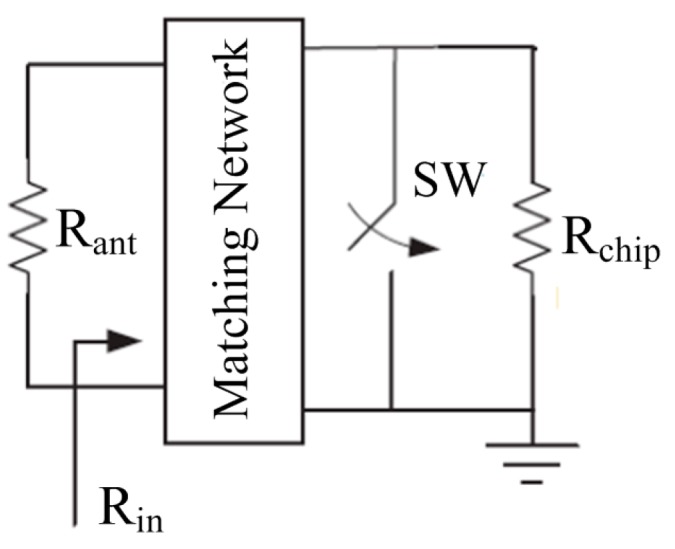
ASK backscattering modulation.

The modulator of the tag chip employs backscattering scheme. Backscattering is a low-power modulation scheme in which the RFID tag acts as a reflector that reflects the incident RF wave back to the RFID reader. Backscattering can be either Amplitude Shift Keying (ASK) or Phase Shift Keying (PSK) backscattering. ASK backscattering is much more simple and efficient than PSK backscattering [13], therefore this work employs an ASK backscattering scheme. In ASK-backscattering modulation (shown in Figure 2), with the switch (SW) closed or open, the chip impedance is varied between perfect match (*R_in_* = *R_ant_*) and complete mismatch (*R_in_* = 0) through the Matching Network, where *R_in_* is the input resistance of the chip and *R_ant_* is the impedance of the antenna.

## 3. Pressure Sensor

Capacitive pressure sensors measure the capacitance between two electrodes with variable separation due to a moving membrane. The basic structure of a capacitor consists of a set of parallel plates or electrodes of area *A*, separated by a distance *d*. The value of the capacitance *C* is given by the following equation [14]:

(1) $$C=\frac{\varepsilon A}{d}$$

where ε is the dielectric constant of the medium between the plates. 

**Figure 3:**
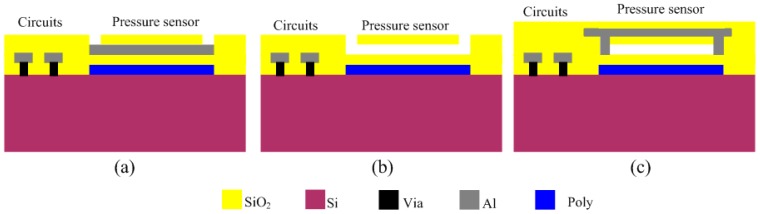
Fabrication processes of the sensor; (**a**) after the completion of the CMOS process; (**b**) etching of the sacrificial lays; (**c**) sealing the etch holes.

**Figure 4:**
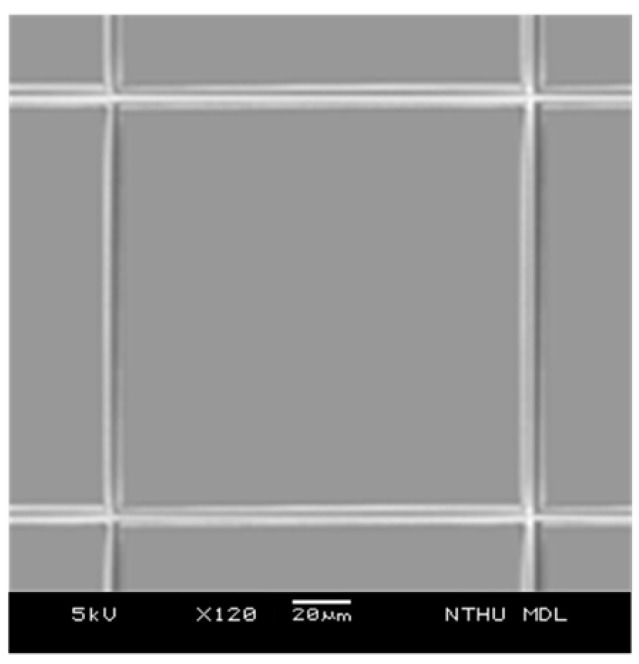
SEM picture of the sensor cells.

The proposed pressure sensor consists of three parts: the top electrode, the dielectric layer and the bottom electrode. The dielectric layer consists of silicon oxide and an air gap. The fabrication process of the sensor is illustrated in Figure 3. It starts with the deposition of polysilicon and patterning to form the bottom electrode at a temperature of 200 °C, which is then covered with a SiO_2_ layer. Aluminum is then sputtered and patterned to form the sacrificial layer, which is also covered with a SiO_2_ layer (Figure 3a). The SiO_2_ layer is patterned to form the releasing hole and then the aluminum layer is released by H_3_PO_4_ at a temperature of 80 °C (Figure 3b). Finally, a 2 μm-thickness aluminum layer is vapored on the surface and patterned to form the top electrode, on which a SiO_2_ layer is then deposited (Figure 3c). The thickness of the sensing gap between the electrodes is about 1.6 μm. These processes in Figure 3a are finished in the CMOS process and other processes are finished in the post-MEMS phase. The total pressure sensor is composed of a 4 × 4 array of sensing cells (shown in Figure 4). All sensing cells have the same structure and dimensions and each sensing cell is a rectangular shape with a side length of 140 μm. 

## 4. Rectifier

The rectifier is based on the Dickson architecture. A popular performance metric of a rectifier is its Power Conversion Efficiency (PCE). The PCE can be defined as follows:

(2) $$PCE=\frac{P_{out}}{P_{in}}=\frac{P_{out}}{P_{in}+P_{loss}}\frac{P_{out}}{P_{in}+NP_{diode}}$$

where *P_in_* is the input power of the rectifier, *P_out_* is the output power of the rectifier, *P_loss_* is the loss of the rectifier, *P_diode_* is the loss of the each switch diode. The *P_diode_* is mainly determined by the turn-on voltage of the diode, therefore, in order to realize large PCE, small turn-on voltage of the diode for reducing forward diode loss is essential [15].

There are several techniques that are used to reduce the turn on voltage, including Schottky diodes [16,17] and low *V_th_* transistors [18], which all require advanced CMOS processing at an additional cost. In standard CMOS technology, different threshold cancellation techniques [19,20,21,22,23] have been proposed for diode-connected MOS transistors to improve the PCE of the rectifier. In this work, a biasing scheme is proposed to enhance the PCE of the differential-drive rectifier [23] for small input voltage/power levels. To achieve a high PCE for small input voltage/power, the boosting scheme has to positively shift the gate voltage of the NMOS switches and negatively shift the gate voltage of the PMOS switches to decrease their respective effective threshold voltage and accordingly increase their forward current.

**Figure 5:**
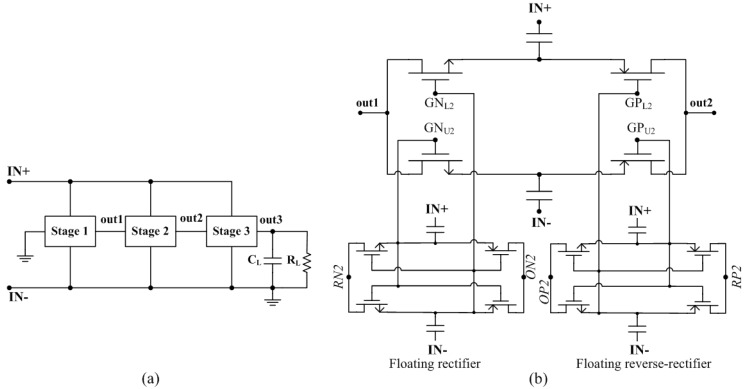
Proposed rectifier; (**a**) entire architecture; (**b**) circuit schematic of the second stage.

Ensuring the enough large supply voltage (>0.8 V) for the sensor interface all the time, the proposed rectifier adopts three-stage architecture (Figure 5a), of which each stage takes the same circuit structure. For the second stage (Figure 5b), the DC input out1 serves as the reference for the floating rectifier (*RN2*) and the DC output out2 serves as the reference for the floating reverse-rectifier (*RP2*). The two floating rectifiers generate the boosted gate drive voltages *GN_L_*_2,*U*2_ and *GP_L_*_2,*U*2_ which are the shifted versions of the RF voltages *M_U_*_2,*L*2_ such that the following conditions are satisfied [24]:

(3) $$GN_{L2}>M_{L2},GN_{U2}>M_{U2},GP_{L2}<M_{L2},GP_{U2}<M_{U2}$$

The output of the floating rectifier (*ON2*) has a higher DC value relative to out2 as it does not drive any load while out2 drives are succeeding stage. Accordingly, the intermediate node voltages of the floating rectifier (*GN_L_*_2,*U*2_) are of a higher DC value compared to (*M_U_*_2,*L*2_). The floating reverse-rectifier is also designed such that the DC component of its intermediate node voltages (*GP_L_*_2,*U*2_) is always smaller than that of the main rectifier (*GN_L_*_2,*U*2_) [24].

## 5. Sensor Interface

The capacitive pressure sensor acts as a capacitor when it works with a sensor interface. There are several popular techniques to perform the capacitance-to-digital conversion function. The universal conversion starts with a capacitance-to-voltage converter followed with a voltage-to-digital converter [25,26,27]. This technique can achieve high speed and high resolution performance. However, due to the use of operational amplifier in Switched-Capacitor Amplifiers (SCA), this technique produces too much power dissipation which is unsuitable for passive designs. Accordingly, the inverter is proposed to replace the operational amplifier in SCA [28,29], which reduces the entire power dissipation significantly. However, they still employ relatively high supply voltage for ultra-low power design. The pulse-width modulation technique [30,31,32] is started with a capacitance-to-time converter followed with a time-to-digital converter. This technique is suitably applied on the field of large-scale capacitance variation. However, it always employs complicated architecture and still consumes high power.

**Figure 6:**
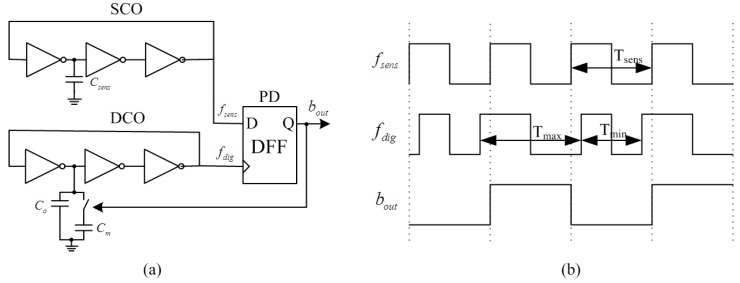
Proposed fully-digital capacitive sensor interface; (**a**) architecture; (**b**) corresponding waveforms for a constant sensor value.

The proposed fully-digital sensor interface (Figure 6a) is based on phase-locked loop theory, which can achieve the capacitance-to-digital conversion directly [33]. It consists of three blocks, including a Sensor-Controlled Oscillator (SCO), Digital-Controlled Oscillator (DCO) and Phase Detector (PD). Both the SCO and the DCO are implemented as three-stage inverter-based ring oscillators. The sensor capacitor (*C_sens_*) acts as the variable load on a single stage of the SCO, thereby generating a sensor-controlled frequency (*f_sens_*). The DCO is steered by the PD output signal (*b_out_*), which is a representation of the phase difference between the SCO and the DCO. The variable capacitive load on a single stage of the DCO consists of two capacitors, *C_o_* and *C_m_*. The capacitor *C_o_*, is designed equal to the quiescent value of *C_sens_*, always connected to the DCO, but the capacitor *C_m_*, designed slightly larger than the maximum variation of *C_sens_*, is swapped in or out of the DCO depending on the feedback from the PD. The PD is simply composed of a single-bit d-flip-flop. The corresponding signals of this sensor interface for a constant sensor value is shown in Figure 6b. When the entire feedback loop is locked, the average digital frequency (*f_dig_*) will correspond to the sensor frequency (*f_sens_*). Therefore, the over-sampled output (*b_out_*) represents the digital value of the sensor capacitance. 

**Figure 7:**
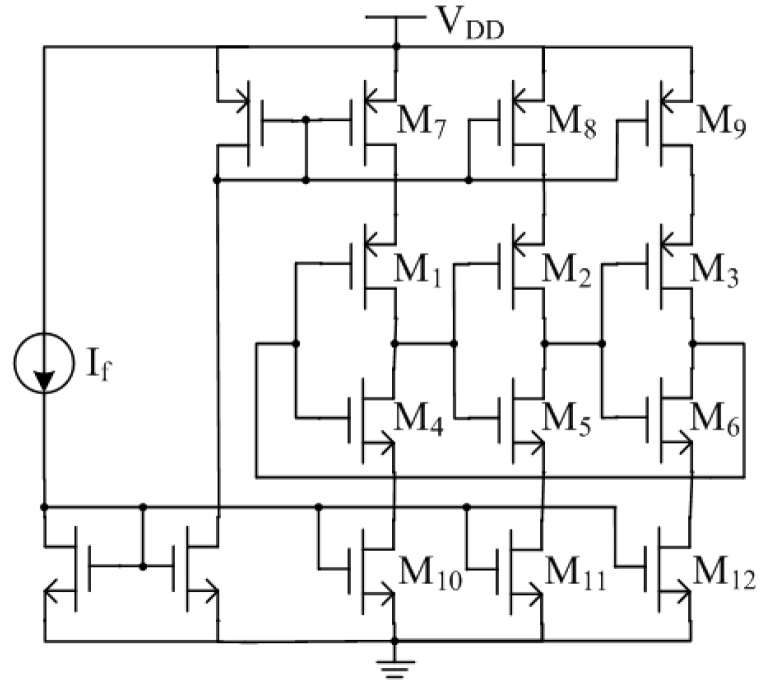
Current-starved ring oscillator.

Compared with the previous design [34], this paper employs current-starved ring oscillators for the design of SCO and DCO. As shown in Figure 7, M_1_–M_6_ form the 3-stages inverter-based ring oscillator, which current is constrained by the current mirror M_7_–M_12_. Although the extra transistors of M_7_–M_12_ need a higher supply voltage, the current flowing through the inverters can be constrained much smaller, resulting in lower power dissipation and higher temperature stability.

Considering the issues of system linearity and process variation, *C_m_* is normally designed as a programmable capacitor. For this work, the pressure sensor’s capacitance varies from 0.8 pF to 1.52 pF within the relative pressure range, hence the capacitor *C_o_* and *C_m_* are selected as 0.8 pF and 0.9 pF, respectively. This architecture has an inherent immunity to supply voltage variations due to the fact that the digital output (*b_out_*) is determined by the phase difference between the DCO and the SCO, which results in the exclusion of a voltage regulator in the proposed passive wireless pressure sensor tag as seen in Figure 1.

## 6. Measurement Results and Discussions

The 0.18 μm CMOS process of Semiconductor Manufacturing International Corporation (SMIC, Shanghai, China) is employed to fabricate the proposed pressure sensor tag. An Agilent 4284 A precision LCR meter (Agilent Technoligies, Santa Clara, CA, USA) was utilized to measure the sensor value. The pressure sensor performances were tested in a pressure chamber. The gas pressure source was provided to the pressure chamber and the gas pressure was tuned by a digital pressure controller. 

**Figure 8:**
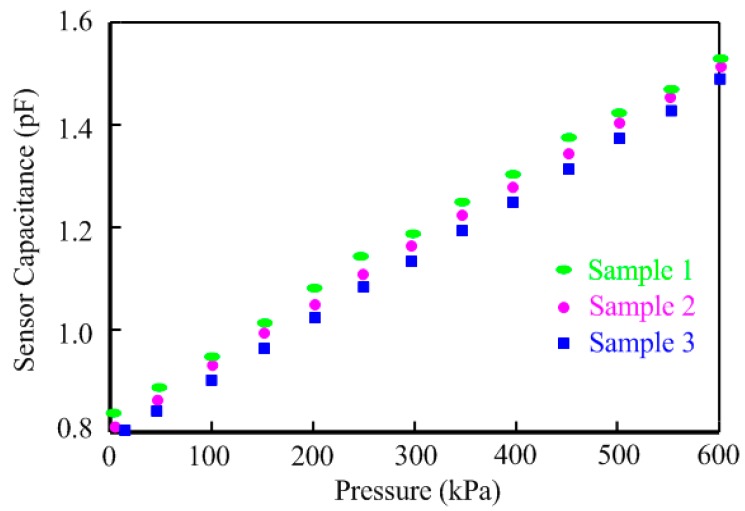
Sensor capacitance *vs.* pressure of three sample chips.

The relationships of three sample chips between the sensor capacitance and the pressure on the sensor are illustrated in Figure 8. These three chips were measured per 50 kPa within the range from 0 to 600 kPa. The proposed pressure sensor achieves excellent linearity with a sensitivity of 1.2 fF/kPa. The three testing results show some deviations and the maximum error in the same pressure does not exceed 9% within the pressure range. The capacitance variations of three sample chips can be contributed to the fabricating process variations. 

**Figure 9:**
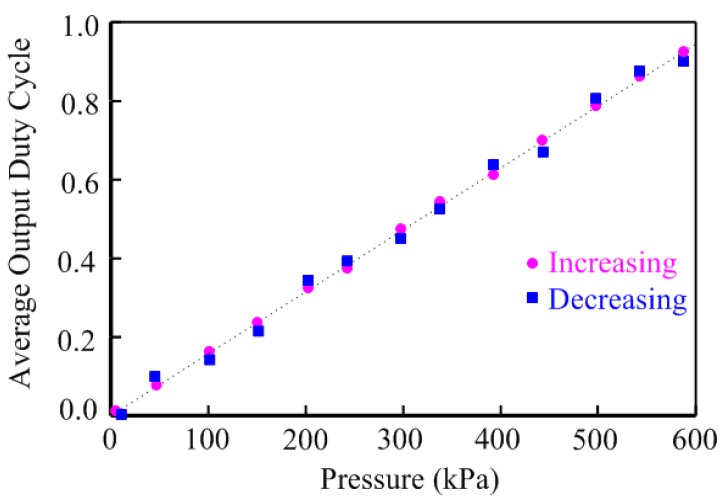
Hysteresis performance of the proposed sensor tag.

In order to test the interface performance, the proposed pressure sensor tag was placed under a constant 0.8 V supply voltage for interface. The sensor tag was measured per 50 kPa increment and then repeated per 50 kPa decrement within the range from 0 to 600 kPa. The various pressures correspond to the various sensor capacitances which then result in the different interface output (*b_out_*). Figure 9 illustrates the relation between the average output duty cycle of the interface and the pressure on the sensor tag. The average output duty cycle of the proposed pressure interface keeps in line with the pressure and shows a little error between the pressure increasing and decreasing.

**Figure 10:**
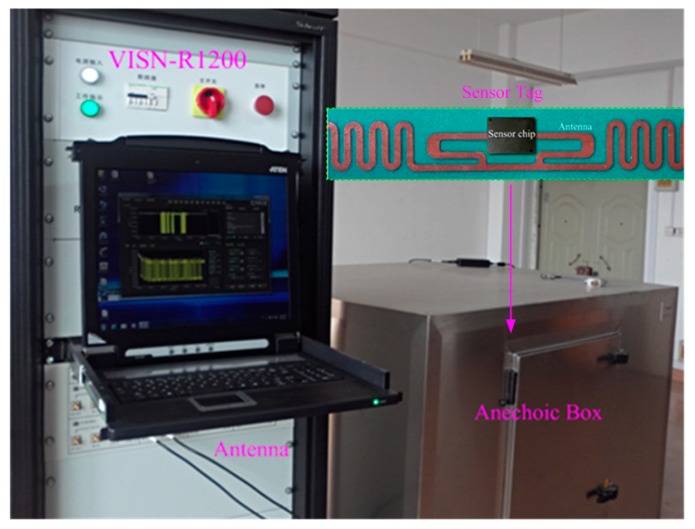
Photo of the wireless sensor tag.

In order to test the wireless performances, the sensor tag chip was matched with a dipole antenna. The wireless test environment consists of a special RFID test instrument, a VISN-R1200 from VI Service Network (city, state abbrev if US, country) and an anechoic box (seen in Figure 10). The VISN-R1200 can work as an adjustable RFID reader and display the testing signals simultaneously. The sensor tag was tested in the anechoic box for the electromagnetic shielding.

**Figure 11:**
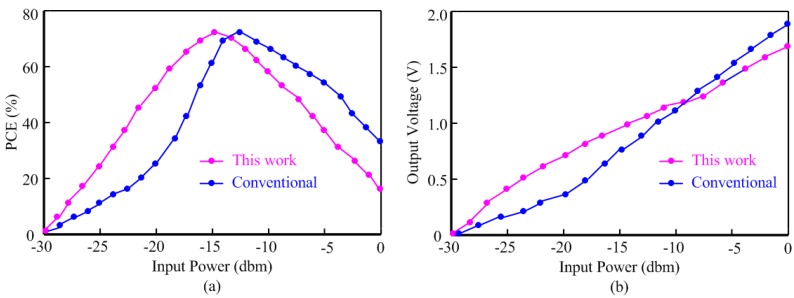
Performance comparisons between conventional architecture and this work; (**a**) PCE comparison; (**b**) output voltage comparison.

For a 900 MHz input frequency, the PCE curve of the proposed rectifier is shown in Figure 11a. Compared to the conventional differential architecture [23], the enhanced efficiency scheme provides a higher PCE for smaller input levels which corresponds to a longer communication distance. The efficiency curve of the proposed rectifier is shifted to the left relative to the conventional rectifier and a PCE of 53% is achieved at the low input power of −20 dBm, while the PCE is 24% for the conventional rectifier at the same input level. The proposed rectifier provides a higher PCE for smaller input levels which corresponds to a longer communication distance. Note that the peak PCE value for the proposed rectifier is slightly lower than that of the conventional rectifier which can be attributed to the power consumption of the floating rectifiers. Figure 11b compares the output voltage of the proposed rectifier with that of the conventional rectifier at 900 MHz input frequency. The enhanced efficiency provides 0.6 V output voltage at the low input power of −20 dBm, while this output for the conventional counterpart is 0.3 V which is insufficient level for many RFID applications.

**Figure 12:**
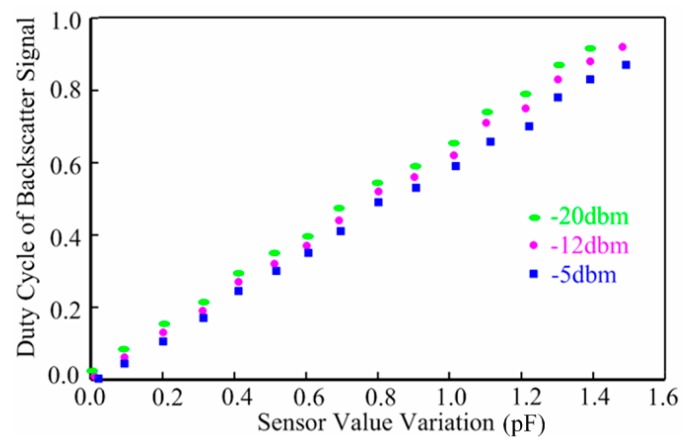
Duty cycle of backscatter signal *vs.* sensor value variation.

The measured minimum power dissipation of the wireless humidity sensor tag is 3.2 µW. The backscattering performance of the sensor tag is shown in Figure 12. Due to the interface’s immunity to the supply voltage, the duty cycle of the backscatter signals achieve a good linearity with the sensor value variations at the different input powers including −20 dBm, −12 dBm and −5 dBm. The slight dependence of the sensor output duty cycle on the input power level is due to the non-ideal PD.

At last, the sensor interface performances comparison with previous designs is shown in Table 1, where FOM can be defined as follows:

(4) $$FOM=\frac{Power}{2^{ENOB}\times 2\times BW}$$

where *BW* is the bandwidth of the interface. The sensor interface performances were measured under a constant 0.8 V supply voltage from the rectifier. Owing to simple architecture, the proposed sensor interface covers reduced chip area and can operate on 0.8 V supply voltage. Although achieving a moderate Effective Number Of Bits (ENOB) and a similar FOM to the reported interfaces in [29,32], the proposed interface reduces power consumption significantly in contrast with the state of the art designs, which is especially suitable for low power application. 

**Table 1:** Performance comparison of integrated sensor interfaces.

Interface	Process (µm)	Supply (V)	ENOB (bits)	Area (mm^2^)	Power (µW)	BW (kHz)	FOM (pJ/conv)
[28]	0.16	1.2	12.5	0.15	10.3	1.25	8300
[29]	0.09	1.0	10.4	N/A	3.0	1.56	1.4
[30]	0.35	3.0	9.3	0.09	54.0	12.60	3.4
[32]	0.32	3.0	9.8	0.52	84.0	2.60	4.5
[34]	0.18	0.5	7.2	0.01	1.1	2.34	1.6
This work	0.18	0.8	7.4	0.01	0.8	2.17	1.5

## 7. Conclusions

This paper proposes a novel monolithic pressure sensor tag for passive wireless applications. It operates in the UHF band and employs an ASK modulator. The pressure sensor element employs CMOS MEMS technology and consists of a 4 × 4 array of sensing cells. A three-stage rectifier achieves high PCE for small input powers by boosting the gate voltage of the switching transistors through a chain of auxiliary floating rectifier cells. The sensor interface, based on phase-locked loop theory, employs fully-digital architecture. Despite the moderate ENOB, this interface can operate on an ultra-low supply voltage and then reduces the power dissipation significantly compared to previous designs. The measurement results show the proposed pressure sensor tag achieves excellent linearity, hysterisis and stability. The rectifier has a PCE of 53% at the low input power of −20 dBm. The total power dissipation of the wireless humidity sensor tag is 3.2 µW. However, this pressure sensor tag doesn’t include the digital components and can’t achieve addressing function, which is the topic of our planned future work. 
